# Building a foundation to promote Women’s health research in Dubai. Study protocol for a project to investigate the feasibility of establishing a dedicated Women’s health biobank

**DOI:** 10.3389/fgwh.2025.1589337

**Published:** 2026-02-12

**Authors:** William Atiomo, Fadi Mirza, Khawla Al Zarooni, Muna Tahlak, Tom Loney, Asiyah Shafi, Osman Zin Al Abdin, Mahmood Al Mashhadani, Samuel Ho, Arnaud Wattiez, Manal AbdulRahim, Mutairu Ezimokhai

**Affiliations:** 1 College of Medicine, Mohammed Bin Rashid University of Medicine and Health Sciences, Dubai Health, Dubai, United Arab Emirates; 2 Obstetrics and Gynecology and Women’s Health, Dubai Health, Dubai, United Arab Emirates; 3 Dubai Health Biobank, Research and Graduate Studies, MBRU, Dubai Health, Dubai, United Arab Emirates; 4 Laboratory Medicine and Pathology, Dubai Health, Dubai, United Arab Emirates

**Keywords:** biobank, endometrial cancer, endometriosis, polycystic ovarian syndrome (PCOS), pre-eclampsia and health research, United Arab Emirates, Women’s health

## Abstract

Women’s health concerns play a role in several of the UAE's major public health challenges, including cardiovascular disease, injuries, cancer, and respiratory conditions. The UAE's 2024 National Policy for improving Women’s health aims to lower cancer mortality rates to 23.24 per 100,000 females, with translational research projects utilizing bio-registries and biobanks supporting this goal. This article describes the protocol for the Dubai Women’s Health Study, a prospective cohort study aimed at investigating the feasibility of establishing a dedicated Women’s health biobank in Dubai to support translational research on improving Women’s health. Initial focus will be on polycystic ovarian syndrome (PCOS), endometriosis, pre-eclampsia, and endometrial cancer, which are prevalent health conditions in the UAE. Recruitment will start at the largest public Women’s hospital in Dubai (Latifa Women’s Hospital), with possible future inclusion of other private hospitals. The study will approach women diagnosed with PCOS, endometriosis, pre-eclampsia, and endometrial cancer over a 12-month period from April 2025 to April 2026, obtaining clinical details and biological samples for establishing a bio-registry and biobank. The feasibility will be evaluated based on recruitment rates, the willingness to contribute samples and logistical challenges. The biobank will support ongoing studies on endometrial cancer risk in PCOS and endometriosis, Co-enzyme A's role in pre-eclampsia, and genetic profiling of inherited endometrial cancer cases in Dubai. The study recruitment only commenced in July 2025 and there are no results yet. Aligned with the National Policy for Improving Women’s Health, the biobank provides a foundation for local and global Women’s health research, addressing historical gender neglect in medical research, and promoting health equity globally.

## Introduction

Women’s health is increasingly recognized as a global public health and development priority, closely aligned with the United Nations Sustainable Development Goals (SDG 3: Good Health and Well-Being and SDG 5: Gender Equality). The United Arab Emirates (UAE) is one of the fastest growing economies globally (1). Dubai, its international hub (2), is composed of a multi-cultural, multi-ethnic society and is home to over 200 nationalities (3). This diverse demography generates a wide range of genomes and phenotypes and a unique opportunity for researchers to study diseases. The UAE Vision 2031 (4) and the 2024 (5) National Policy for Improving Women’s Health emphasize innovation, precision medicine, biobanking, and data-driven policy to improve health outcomes.

On the 5th of February 2024, His Highness Sheikh Mohammed bin Rashid Al Maktoum, Vice President and Prime Minister of the UAE, and the Ruler of Dubai approved the National Policy for Improving Women’s Health in the UAE (5). The policy aims to develop a multisectoral national framework to promote Women’s health by providing high quality and efficient health services whether curative, preventive or rehabilitative. The policy specifically aims to reduce the cancer mortality rate from 70.3 to 23.24 and the all-cause mortality rate to 62.77 per 100,000 of the female population (6). Additionally, the policy's targets include reducing health issues arising from unhealthy lifestyles (e.g., lack of physical activity, poor diets, and obesity) by 3% (5). Implementing these agendas requires high-quality research across diverse Women’s health issues. Such research is essential for evidence-based policy and clinical decision-making. Women’s health issues contribute to some of the “Big 4” public health issues in the UAE (cardiovascular disease, injury, cancers, and respiratory diseases) (7). For example, women with polycystic ovary syndrome (PCOS) are at increased risk of cardiovascular disease, endometrial cancer (EC) (39), and asthma (8). Women who develop pre-eclampsia during pregnancy are at increased risk of cardiovascular disease in later life (9, 10) and women with endometriosis may also be at higher risk of breast and endometrial cancer (EC) (11). Of concern, is that there might be a higher prevalence of risk factors and Women’s health conditions in the UAE population compared to the global prevalence (12); however, population-based research is required to provide accurate and reliable estimates to make these comparisons and inform healthcare planning and expenditure in the UAE.

Recently, Dubai Health, the newly formed Academic Healthcare System, established a world-class biobank to support translational research. Dubai Health is Dubai's first integrated academic health system established to elevate the standard of care and to advance health for humanity. Dubai Health (13) is comprised of 6 hospitals, 26 ambulatory health centers and 21 medical fitness centers, Mohammed Bin Rashid University of Medicine and Health Sciences and Al Jalila Foundation. The mission is to serve patients through the integration of care, learning, discovery and giving. The family of 11,000 collaborates across multidisciplinary teams to put the patient first. Dubai Health Biobank is one of the largest in the country, having the capacity to store up to seven million biological samples. This provides a unique opportunity to support research into Women’s health in the UAE and provide collaboration opportunities with international researchers. Well-designed bio-registries and biobanks play a pivotal role in advancing health research. They enable longitudinal insights into disease risk, precision medicine, biomarker discovery, and epidemiology (14). This research infrastructure is therefore crucial to achieving the goals of the National Policy for Improving Women’s Health in the UAE.

Outside of Dubai, within the UAE, biobanking capacity has also expanded in recent years, with other initiatives such as the Abu Dhabi Biobank (15) providing high-quality biospecimen storage linked to clinical data. These platforms will support a wide range of biomedical research; however, they are largely population-based with no dedicated national biobank focused specifically on Women’s health conditions. Across the wider Gulf region, major population biobanks have been established, including the Qatar Biobank and the Saudi Biobank. These initiatives include substantial female participation. However, their primary focus remains general population health, genetics, and non-communicable diseases, with limited emphasis on women-specific conditions. A systematic review published in 2020 (16) identified 81 maternal and birth cohort studies from six Gulf Cooperation Council (GCC) countries (Bahrain, eight in Kuwait, seven in Qatar, six in Oman, 52 in Saudi Arabia, and seven in the UAE) primarily focused on correlation between maternal/reproductive and medical exposures, with birth and maternal outcomes. However, many studies were descriptive, underscoring the need for more robust long-term cohorts covering broader exposures. In the MENA region, Women’s health research is often conducted through disease-specific registries, hospital-based cohorts, or short-term studies rather than through dedicated, longitudinal Women’s health biobanks, and where a biobank is present, face challenges related to population level long-term biobank infrastructure (17). Globally, several large cohorts—including the Nurses' Health Study, the Women’s Health Initiative, the UK Biobank (18), and the All of Us Research Program (19)—have demonstrated the scientific value of linking biospecimens with longitudinal clinical data and enabling sex-stratified analyses. However, most global resources are not designed as dedicated Women’s health biobanks and predominantly reflect Western populations, underscoring the need for regionally anchored initiatives that address ethnic, genetic, and environmental diversity.

The Dubai Women’s Health Biobank aims to addresses these gaps by generating women-specific, ethnically diverse data, directly supporting SDG 3 (Good Health and Well-Being) and SDG 5 (Gender Equality). However, as establishing a biobank requires substantial infrastructure, governance, and stakeholder engagement, a feasibility study becomes a critical first step to determine operational viability. The International Society for Biological and Environmental Repositories (ISBER), (20) explicitly recommends that feasibility assessments precede implementation to determine the practicality of collection processes, infrastructure adequacy, staffing, and financial sustainability. It highlights how feasibility studies identify potential operational barriers early, support compliance with biospecimen quality standards, and ensure that planned collections align with scientific demand, thereby reducing risk and improving reproducibility. The United States National Cancer Institute also emphasizes the critical role of feasibility evaluation before establishing any biospecimen resource (21). It demonstrates how conducting feasibility analyses ensures that biobank objectives are realistic, ethically sound, and economically viable, while minimizing waste of resources and avoiding premature implementation of non-sustainable infrastructures. Finally, Soo et al. (22) describe the practical outcomes of performing a feasibility study before creating an academic biobank in a low-resource context and how early technical and financial assessments guided phased implementation, ensuring alignment between goals and capacity.

A feasibility study is more than planning. It safeguards scientific integrity, ensures ethical compliance, and supports financial accountability in biobank establishment. This article describes the protocol and progress so far with a study to investigate the feasibility of developing a dedicated Women’s health biobank in Dubai (UAE) to support research to improve Women’s health in the region. The objectives cover two areas. First, biobank operations: building data infrastructure, linking health records with samples, and measuring recruitment rates and willingness to contribute. Second, Women’s health conditions: collecting blood and stool samples to study EC risk in PCOS and endometriosis, the role of co-enzyme A in pre-eclampsia, and inherited EC cases in Dubai. The study also aims to evaluate logistical challenges (which is sample transportation, storage), measure financial sustainability for long-term projects, and identify the ethical and legal compliance requirements.

## Methods and analysis

### Study design

**Figure 1:**
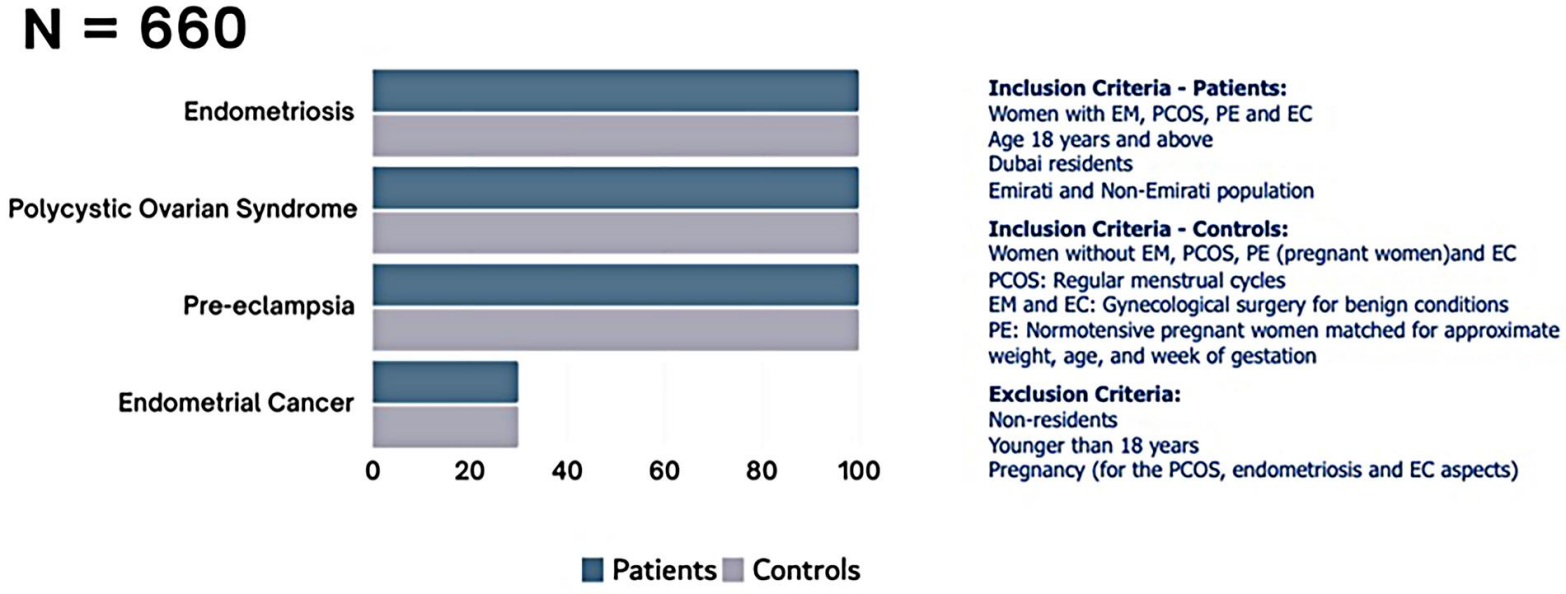
Women’s health conditions of initial focus in the dubai Women’s health biobank.

**Figure 2:**
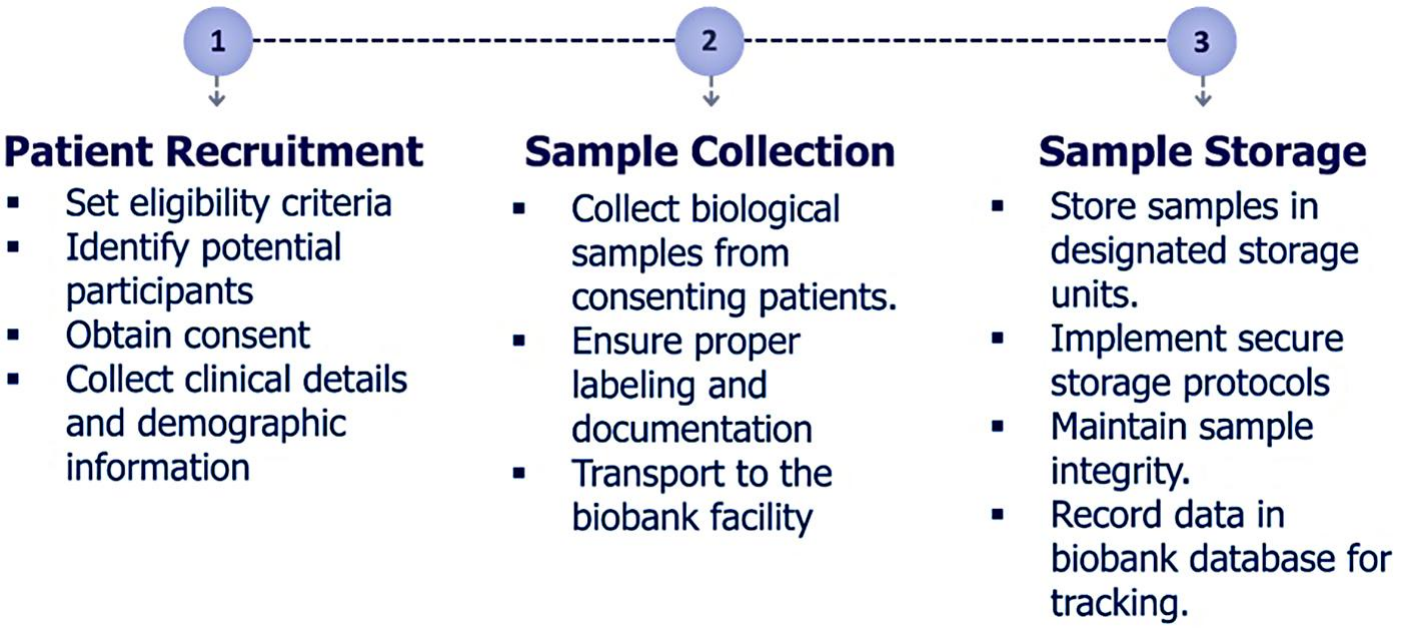
Study design. The Dubai Women’s Health Study will be a prospective cohort study in Dubai, United Arab Emirates.

**Figure 3:**
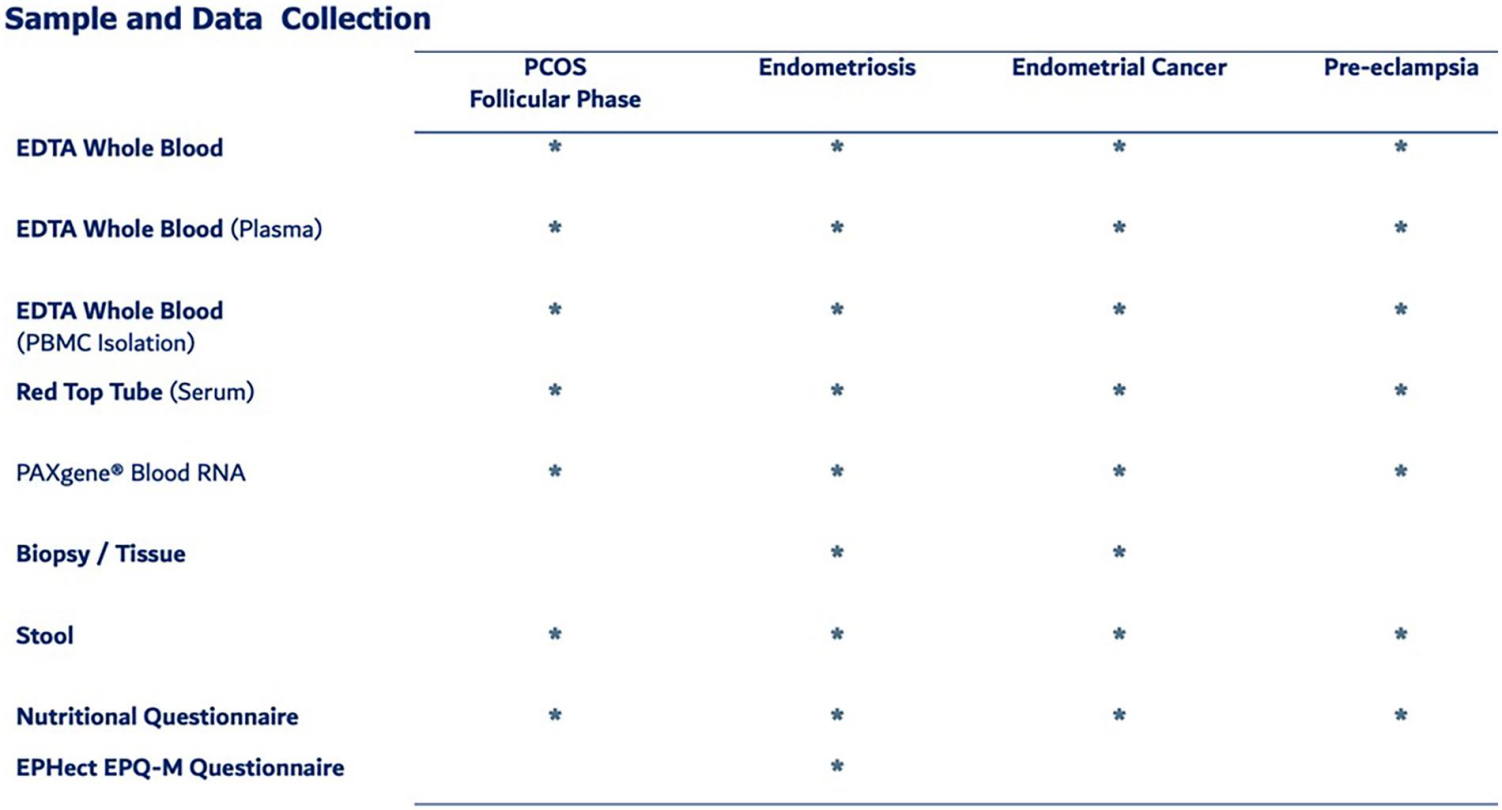
Dubai Women’s health study sample and data matrix.

**Figure 4:**
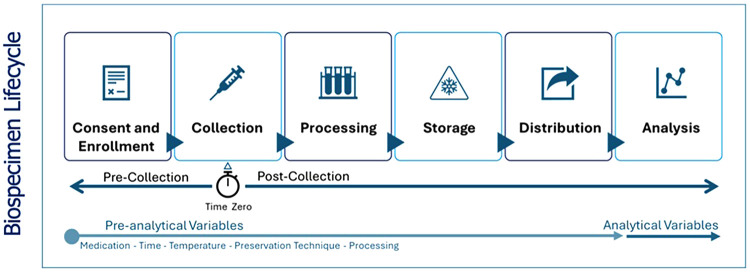
Controlled biospecimen handling throughout its lifecycle to ensure high-quality, reliable samples for research.

**Figure 5:**
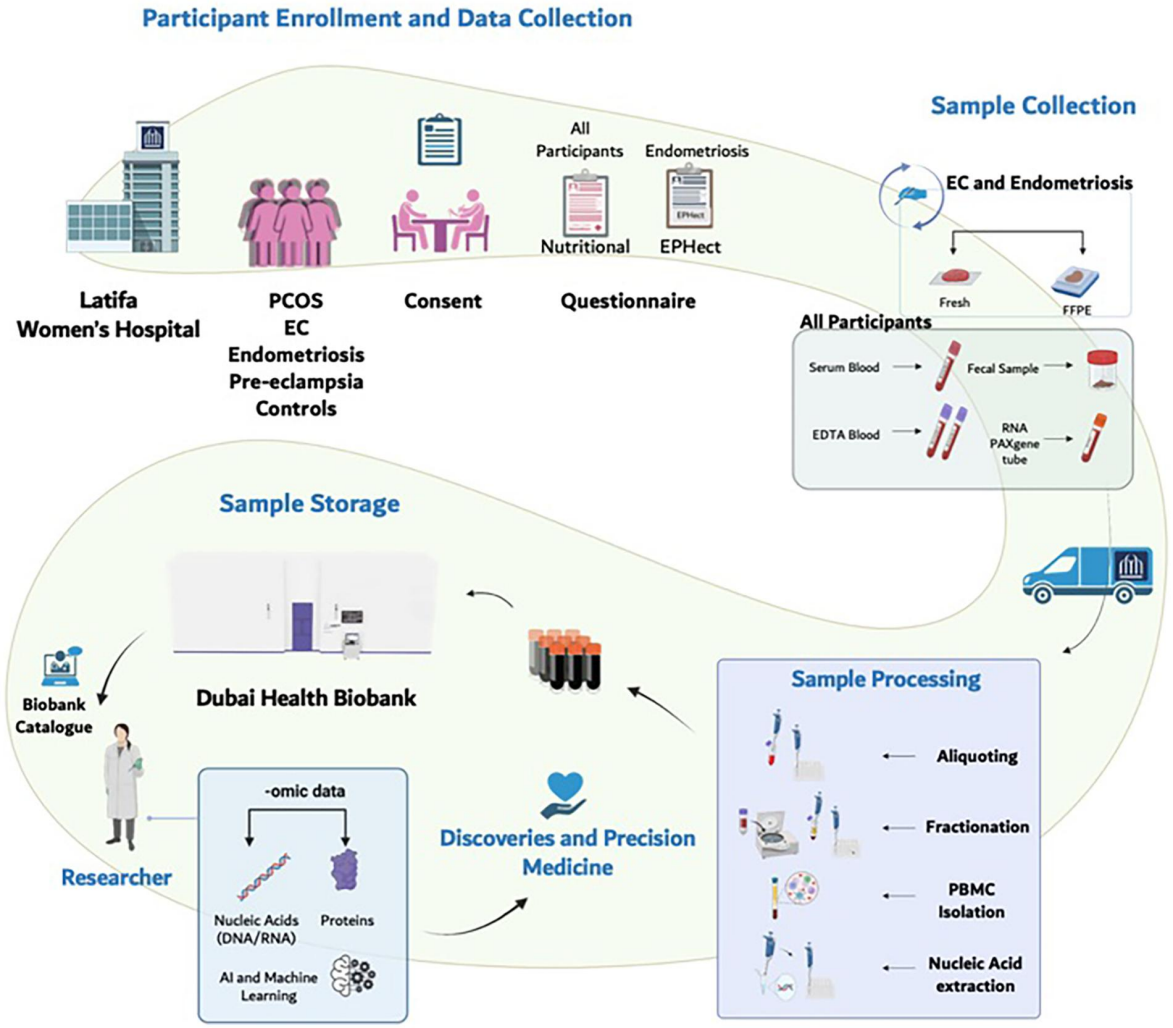
Workflow of the dubai Women’s health study.

The Dubai Women’s Health Study will be a prospective cohort study in Dubai, UAE. The conditions of initial focus will include the following common and important Women’s health conditions in the UAE: PCOS, endometriosis, pre-eclampsia, and EC (Figure 1). Recruitment will start at Latifa Women’s Hospital in Dubai and may include other hospitals in the future. Latifa Women’s Hospital is part of Dubai Health, a public institution providing free or subsidized and accessible care to Emirati and non-Emirati citizens residents. This ensures broad socioeconomic representation. Latifa Hospital is a tertiary center that manages all four target conditions—PCOS, endometriosis, pre-eclampsia, and endometrial cancer—making it an ideal site for recruitment. The campaign will focus on key staff such as surgeons, specialists and nurses in obstetrics and gynaecology. Patient recruitment, sample collection and sample storage (Figure 2) are outlined below. The sample and data matrix are illustrated in Figures 3, 4. Figure 4 shows controlled biospecimen handling throughout its lifecycle to ensure high-quality, reliable samples for research. Figure 5 shows the workflow.

### Participants

#### Inclusion criteria

The study aims to approach all women seen and given a diagnosis of endometriosis, PCOS, pre-eclampsia and EC over a 12-month period from April 2025 to April 2026 at Latifa Women’s hospital to seek consent to obtain clinical details and biological samples into the Biobank Information Management System (BIMS). Women approached will include all nationalities, be aged at least 18 years, resident in Dubai and able to provide informed consent. We will also aim to recruit women without endometriosis, PCOS, pre-eclampsia (pregnant women) and EC as controls. Control women for PCOS will be women with regular menstrual cycles identified from a gynecology or other appropriate outpatient setting. Control women for endometriosis and EC will be women undergoing gynecological surgery for benign conditions other than endometriosis or EC. Control women for pre-eclampsia will be normotensive pregnant women matched for approximate weight, age (5-year age group), and week of gestation.

#### Exclusion criteria

The study will exclude the following women. Non-residents in Dubai as they are less likely to be available for long-term follow-up, pregnancy (for the PCOS, endometriosis and EC aspects), younger than 18 years, and those unable to provide consent.

#### Participant recruitment

Institutional review board (IRB) approval from the MBRU IRB in Dubai was obtained in February 2025 (MBRU IRB-2024-692). In collaboration with gynaecologists, obstetricians, oncologists, and other healthcare providers (nurse or physician) eligible women will be identified and approached by an on-site trained research assistant with an information sheet detailing the project. Diagnosis will be based on the clinical diagnosis made by the physician supported by the relevant ICD-10 code. In women with PCOS, this will be verified by the Principal Investigators of the study, given previous research demonstrating disparity between physician and electronic health record diagnosis of PCOS and objective diagnosis using the Rotterdam, National Institutes of Health (NIH) or androgen excess and PCOS (AE-PCOS) criteria (40). Pre-eclampsia will be defined as the onset of a new episode of hypertension during pregnancy, characterized by persistent hypertension (diastolic blood pressure ≥90 mm Hg) and substantial proteinuria (>0.3 g/24 h or more or protein level of +2 or above in the urine dipstick test) (23).

If women express interest to participate, written informed consent will be obtained. This consent will include permission for follow-up interviews, extraction of health information from medical records, collection, and long-term storage of biospecimens and future research into the mechanisms of disease (including genetics) in the health conditions of interest and assurance of confidentiality. The broad consent model supports the collection of specimens and data for future, unspecified use within a broad set of parameters (24). The study will be conducted in accordance with the principles of the Declaration of Helsinki (25). Each participant will have the right to withdraw from the study at any time without giving any reason. During enrolment, the participant will be provided with a detailed information sheet on the study description, contact details of the research team (telephone number and email address) and the withdrawal process.

### Data and sample collection

#### Clinical data

We will collect relevant clinical data including demographics (e.g., age, ethnicity, education level, occupation status and type), reason for clinical presentation, menstrual history, date of last menstrual period before sample collection (if appropriate), past medical or surgical history, medication, allergies, and smoking history from the patient and medical records, The most recent height, weight, and blood pressure measurements in the electronic medical record will be recorded. The details of the last pelvic ultrasound scan will also be recorded to confirm if the ovaries did or did not look polycystic in women with PCOS. The clinical diagnosis (endometriosis, PCOS, pre-eclampsia or EC) and the disease stage (or phenotype) and grade will be recorded. Treatment history will be recorded and a comprehensive database linking clinical information to sample identifiers will be maintained. The research database/bio registry will be linked to biospecimens using the medical record number and unique identification number assigned to each participant when consenting to the study. We will leverage the Biobank Information Management System (OpenSpecimen) for the seamless capture of specimen and research data, while ensuring integration with the Epic electronic health record system, to facilitate efficient data management. This will also enable us to capture any additional variables required to measure socioeconomic status and information about nationality of participants and how long they have been residents of Dubai, as the quality of health care in previous residences, may influence the degree of severity, of the health conditions of interest. Patient privacy and confidentiality will be protected by removing personally identifiable details such as names and dates of birth from samples and health data before they are shared with researchers. Instead, each sample will be assigned a unique code, keeping the patient data anonymous. The Dubai Health Biobank will label all samples and data with this code, ensuring that they cannot be traced back to individuals. Strict security measures such as data encryption and access controls will be put in place to restrict access, thereby allowing only authorized personnel to handle sensitive information in the electronic database.

#### Blood and tissue

For women with PCOS and controls, whole blood (for peripheral blood mononuclear cells (PBMCs), serum and plasma will be collected, for women with endometriosis and their controls, whole blood, serum, plasma, peritoneal and endometrial biopsies will be collected at surgery and for women with EC and their controls, whole blood, serum, plasma and endometrial biopsies will be collected. For women with pre-eclampsia and their controls, whole blood, serum and plasma will be collected in this feasibility study but will include placenta and cord blood in future studies. We will aim to collect the samples at the same time as there is a clinical indication to obtain these samples as part of their routine clinical care. Stored blood samples will be analyzed for hormone profiles and metabolic parameters such as glucose and insulin. These tests will be performed when not already part of routine clinical care. We recognize that obtaining endometrial and peritoneal biopsies from control participants might be challenging, however this will be an outcome measure in this project. We will collect PCOS samples during the follicular phase of the menstrual cycle. We will also assess the challenges of identifying this phase, since many women with PCOS are anovulatory.

Sample collection and storage will comply with the robust standard operating procedures of the Dubai Health biobank and following International Society for Biological and Environmental Repositories (ISBER) best practices for repositories. Briefly, for whole blood, serum and plasma, venous blood samples will be obtained using standard phlebotomy techniques and appropriate anticoagulants (e.g., EDTA) for whole blood preservation and we will label samples with unique identifiers. A separate tube, from the same venipuncture site will be used to obtain blood to be processed for serum and plasma. For peritoneal biopsies, samples will be obtained under sterile conditions during laparoscopy or laparotomy and biopsies obtained from affected areas (endometriosis) and non-affected areas will be snap frozen fresh in liquid nitrogen or in formalin or other suitable fixatives, placed in sterile containers for transport and storage. Samples will be labelled and stored properly. EC biopsies will be obtained during cancer surgery for patients with EC or for controls with benign gynaecological conditions, endometrial sampling will be obtained using aspiration biopsy, curettage of the uterine cavity or hysteroscopic guided endometrial sampling as needed. Samples will be snap frozen fresh in liquid nitrogen or in formalin or other suitable fixatives, labelled and transported to the Dubai Health biobank.

#### Stool samples and nutritional assessment

All patients and controls will also be asked to provide a stool sample in a sterile stool collection container. Stools will be collected using current optimal protocols (26) in the biobank. First, the stool sample will be aliquoted and stored at −20 °C, in addition, one aliquot will be collected in RNAlater and stored at −80 °C, which has been shown to provide stable long-term storage (27). In addition, the patient will complete a questionnaire about their diet and food consumption using a food frequency questionnaire, which will provide information about both recent and habitual diet. In this feasibility study, we aim to use a widely used and validated food frequency questionnaire (FFQ) available at General Documentation Nutrition Questionnaire Service Center Harvard T.H. Chan School of Public Health. This has been validated previously (28). However, we will revise the questionnaire in the main study, to include culturally relevant questions on physical activity and activities of daily living, adapting validated regional instruments.

### Biobank storage and management

The Dubai Health Biobank comprises an Azenta BioStore automated storage system for managing and storing samples at −80 °C. Blood and fecal samples will be processed and stored at −80 °C in the biobank according to established protocols (Figure 3). These processes will involve blood fractionation, DNA and RNA extraction. Tissue processing and embedding, block microtomy and cryo-sectioning and hematoxylin and eosin immunohistochemistry will be handled at the histopathology laboratory at Dubai Health. For long term storages, tissue samples will be stored in formalin-fixed paraffin-embedded (FFPE) blocks at room temperature in the Dubai Health Biobank. The Dubai Health biobank will regularly monitor sample integrity and quality. A Biobank data governance committee (the Biobank Scientific Advisory Committee) will be created to develop protocols for data entry, quality control, privacy protection and access by independent researchers in future research collaborations.

Funding for this project was successfully secured following a competitive bid from the Dubai Health, Collaborative Stimulus Research Grant (CSRG) in October 2024 (CSRG-24-10). The scheme was set up to support funding to stimulate better collaborative research among investigators at Dubai Health clinical entities and MBRU, enhancing the academic mission to promote translational/clinical research collaborations at Dubai Health. Following the award of the grant, a kickoff meeting of the project team was held on October 21, 2024, during which the overview of the project was reviewed, and team roles assigned. A project plan has been developed to define the work breakdown, structure and list the major work packages, deliverables, tasks, and milestones of the project. A project manager started in January 2025 and institutional review board (IRB) approval was obtained in February 2025 (MBRU CSRG-24-10). Standard operating procedures, a communication and patient engagement strategy as well as plans for successful system integration of data stored in the Dubai Health Biobank as part of the Dubai Women’s health study with the Dubai Health Epic medical record system are also currently underway. Following these activities, the next steps would be patient recruitment, data collection and sample biobanking, data analysis and reporting. The study recruitment commenced in July 2025 and there are no results yet.

### Follow-up collaboration and data sharing

Following ethical approval from relevant authorities and institutional review boards, follow-up visits with participants to collect additional samples (if needed) and to update clinical information may be arranged. We will also aim to collaborate with other local and international research institutions and biobanks for data sharing and collaborative studies, ensuring compliance with data protection regulations.

### Patient involvement

We will convene a focus group drawn from a Women’s health advocacy group to obtain structured feedback on participant-facing materials, including informed consent documents, recruitment videos, and proposed engagement strategies. Insights from this focus group will directly inform refinements to study materials and recruitment approaches, ensuring they are acceptable, culturally appropriate, and responsive to Women’s priorities and concerns. In addition, participants will contribute verbatim qualitative feedback during the recruitment process regarding their motivations for participation, perceived barriers, preferences for follow-up, and suggestions to improve recruitment strategies. This feedback will be collected through structured free-text fields and brief documented interviews conducted by trained research staff. Recruitment will use multiple strategies: clinician referral, on-site research assistants, culturally appropriate information sheets, and multilingual consent materials. These approaches aim to maximize inclusivity across Dubai's diverse population. These combined approaches strengthen patient involvement by embedding participant perspectives directly into recruitment design and feasibility evaluation. Participants will not be routinely informed about study outcomes unless there are any discoveries made that have serious and important health consequences for participants or their families.

### Sample size

The purpose of this study is to determine the feasibility of gathering adequate samples for prospective biomarker studies. It is the goal for this pilot study to attempt to recruit women with endometriosis (*n* = 100), polycystic ovary syndrome (*n* = 100), pre-eclampsia (*n* = 100) and EC (*n* = 30). The goal is to be as inclusive as possible in terms of the numbers of patients to serve as a resource for diverse researchers. For each study group we will aim to recruit an equivalent number of age matched controls. Identifying 100 eligible subjects for endometriosis, pre-eclampsia, and PCOS will allow us to estimate a 50% participation rate with 95% confidence within ±10% (29). Although our initial recruitment site will be Latifa Hospital in Dubai, we will extend recruitment to other hospital sites in Dubai if needed. The selected sample size for this pilot study was based on achieving objectives and practical considerations such as feasibility, available resources, and time constraints. Due to the exploratory nature of the study and the need to assess the study procedures and measures, a smaller sample size was deemed sufficient to provide valuable insights and inform decisions about the potential for conducting a larger study in the future. Although a formal sample size calculation was not conducted, the chosen sample sizes are expected to yield valuable preliminary data and facilitate the refinement of study protocols and procedures that can be used to design larger prospective studies testing specific hypotheses.

### Outcome measures

We will evaluate the feasibility of developing a Women’s Health biobank in Dubai based on; participant recruitment rate including the willingness to contribute samples and questionnaires; logistical challenges (sample transportation, storage); costs of sustaining a dedicated Women’s health biobank, and the availability of sufficient samples to conduct the baseline studies of interest. Following the demonstration of feasibility, we plan to broaden recruitment to encompass additional key Women’s health conditions and establish longitudinal follow-up cohort studies to advance personalized medicine. Detailed clinical phenotyping will be conducted, followed by genetic analyses aimed at identifying both genetic predispositions within the population and modifiable risk factors. For example, this will include the assessment of perinatal mental health using the Depression, Anxiety, and Stress Scale (DASS) questionnaire. The study recruitment only commenced in July 2025 and there are no results yet.

### Reporting statement

This study protocol is reported in accordance with the CONSORT 2010 extension for pilot and feasibility studies, the STROBE guidelines for observational cohort studies, and the STROBE-ME guidelines for molecular epidemiology research (Table 1).

**Table 1:** This table maps reporting items from CONSORT 2010 extension for pilot and feasibility studies, STROBE, and STROBE-ME to the corresponding sections of the manuscript: building a foundation to promote Women’s health research in dubai.

CONSORT 2010 extension for pilot and feasibility studies		
CONSORT item	Description	Manuscript location
1a	Identification as a pilot/feasibility study in title	Title
1b	Structured abstract with feasibility objectives	Abstract
2a	Scientific background and rationale for feasibility	Introduction (paras 1–6)
2b	Specific feasibility objectives	Introduction (final paragraph); Outcome Measures
3a	Description of pilot study design	Methods—Study Design
3b	Changes to methods after commencement	Not applicable (protocol; recruitment ongoing)
4a	Eligibility criteria	Methods—Participants (Inclusion/Exclusion Criteria)
4b	Settings and locations	Methods—Study Design; Participants
5	Interventions (if applicable)	Not applicable (observational feasibility study)
6a	Prespecified feasibility outcomes	Methods—Outcome Measures
6b	Changes to outcomes	Not applicable
7a	Rationale for sample size	Methods—Sample Size
7b	Interim analyses or stopping rules	Not applicable
8	Randomisation	Not applicable
9	Allocation concealment	Not applicable
10	Implementation	Not applicable
11a	Blinding	Not applicable
12	Statistical methods (descriptive)	Methods—Outcome Measures; Sample Size
13	Participant flow	Figure 5; Methods—Participant Recruitment
14	Recruitment dates	Abstract; Methods—Study Design
15	Feasibility outcomes estimation	Methods—Outcome Measures
16	Ancillary analyses	Not applicable (future studies described)
17	Harms or unintended effects	Not applicable (feasibility protocol)
18	Limitations specific to feasibility	Discussion (Limitations paragraphs)
19	Generalisability to future study	Discussion; Conclusion
20	Interpretation in context of feasibility aims	Discussion; Conclusion
21	Ethics approval	Ethics and Dissemination
22	Registration	Not applicable (protocol; not a clinical trial)
23	Funding	Funding section
STROBE Checklist—Observational Cohort Study		
STROBE Item	Description	Manuscript Location
1a	Study design in title/abstract	Title; Abstract
1b	Informative abstract	Abstract
2	Background and rationale	Introduction
3	Objectives and hypotheses	Introduction; Outcome Measures
4	Study design	Methods—Study Design
5	Setting	Methods—Study Design
6a	Eligibility criteria	Methods—Participants
6b	Methods of follow-up	Methods—Follow-Up Collaboration
7	Variables	Methods—Data and Sample Collection
8	Data sources and measurement	Methods—Clinical Data; Questionnaires
9	Bias	Methods—Participant Recruitment; Discussion (Limitations)
10	Study size	Methods—Sample Size
11	Quantitative variables	Methods—Clinical Data
12a	Statistical methods	Methods—Outcome Measures
12b	Subgroups and interactions	Not applicable (feasibility)
12c	Missing data	Not applicable (protocol stage)
12d	Loss to follow-up	Methods—Participants; Follow-Up Collaboration
12e	Sensitivity analyses	Not applicable
13a	Numbers at each stage	Methods—Participant Recruitment; Figure 5
13b	Reasons for non-participation	Outcome Measures (feasibility metrics)
13c	Flow diagram	Figure 5
14a	Descriptive data	Planned (Outcome Measures)
14b	Missing data	Not applicable
15	Outcome data	Not applicable (no results yet)
16	Main results	Not applicable
17	Other analyses	Not applicable
18	Key results	Not applicable
19	Limitations	Discussion
20	Interpretation	Discussion; Conclusion
21	Generalisability	Discussion
22	Funding	Funding section
STROBE-ME Checklist—Molecular Epidemiology/Biobanking		
STROBE-ME Item	Description	Manuscript Location
1	Study design and use of biospecimens	Title; Abstract; Methods—Study Design
2	Rationale for biomarker use	Introduction; Objectives
3	Participant selection for biospecimens	Methods—Participants; Recruitment
4	Biospecimen type and anatomical site	Methods—Blood and Tissue; Stool Samples
5	Collection procedures	Methods—Data and Sample Collection
6	Timing of collection	Methods—Blood and Tissue
7	Processing procedures	Methods—Sample Collection and Storage
8	Storage conditions	Methods—Biobank Storage and Management
9	Number of freeze–thaw cycles	Not applicable (automated −80 °C storage described)
10	Laboratory assays	Planned (Methods—Blood and Tissue)
11	Assay validity and reliability	Referenced via ISBER standards
12	Batch effects	Not applicable (feasibility stage)
13	Biospecimen governance	Methods—Biobank Storage and Management
14	Ethical approval and consent	Ethics and Dissemination; Methods—Recruitment
15	Data linkage	Methods—Clinical Data; BIMS/Epic integration
16	Data sharing	Follow-Up Collaboration and Data Sharing
17	Limitations related to biospecimens	Discussion

## Discussion

The Dubai Women’s Health Study will provide high-quality biological samples. These samples can support translational research, help achieve UAE national health targets, advance global research, and accelerate progress toward the UN Sustainable Development Goals (5). Biobanks and bioregistries are an important foundation for modern biomedical research, serving as key infrastructures for advancing discovery, understanding diseases, and developing treatments. Biobanks facilitate research in genomics and personalized medicine by providing samples for studying genetic factors, environmental influences, and disease mechanisms. Bioregistries complement biobanks by integrating patient data, including clinical information, allowing researchers to track disease progression, monitor treatment outcomes, and conduct epidemiological studies. Together, they enable targeted research in fields such as cancer, cardiovascular diseases, and neurodegenerative disorders.

These infrastructures also support collaboration and open science by promoting data and sample sharing across institutions (30) and fostering large-scale studies, such as those seen in the UK Biobank (18) and the All of Us Research Program (19). They are instrumental in precision medicine, where understanding genetic and environmental factors aids in developing tailored personalized treatments. Additionally, biobanks must adhere to ethical standards, ensuring patient privacy and consent while maintaining regulatory compliance. Examples, such as the Cancer Genome Atlas (41) and the Copenhagen City Heart Study (31), show how biobanks advance disease research and treatment. They provide insights that improve precision healthcare and patient outcomes worldwide. The world revenue for biobanking is also expected to exceed $53 billion by 2027 (30), indicating the growing importance of this sector. Additionally, promoting wider representation in global health research can lead to more inclusive and effective outcomes. Dubai's unique multicultural composition, coupled with good access to the Emirati population, provides a valuable opportunity for diverse and impactful research initiatives in the region.

There was discussion about when to administer questionnaires and food diaries. Timing will depend on the patient's condition, since one approach may not suit all four health conditions. We will, however, aim to complete the nutrition questionnaires at the same time as the stool collection. The current plan for now is that de-identified data relevant to the study will be collected on Microsoft Forms with a bulk upload of the data onto a database to be stored in the Biobank Database housed in the Al Jalila Foundation building in Dubai. With respect to endometriosis patients, the EPHECT endometriosis questionnaire was initially deemed overly complicated for this feasibility study. A simpler questionnaire was therefore recommended for now, with the EPHECT questionnaire possibly used in a follow-up study due to its validation and standardization. However, some colleagues encouraged using the EPHECT questionnaire sooner to foster collaboration with international endometriosis groups. Contact was therefore established with the Endometriosis Phenome and Biobanking Harmonisation Project (EPHect) (32), who have been helpful, including providing us with access to a validated Arabic version of the EPHECT questionnaire. We also intend to register our center with EPHect following IRB approval. This is important in the context of recent calls to scale up collaboration and data sharing in cohort studies (33). We also intend to establish collaborations with international groups with regards to patients recruited into the other Women’s health conditions (PCOS, pre-eclampsia and EC) of initial interest in our study.

Establishing a dedicated Women’s health biobank is complex. It involves ethical, legal, and operational challenges. One consideration is the alignment of local (UAE) and international ethical standards (34). The World Medical Association (WMA) declaration of Taipei on ethical considerations regarding health databases and biobanks (2016) (35) outlines fundamental principles, including the need for voluntary participation and the assurance of confidentiality in the collection, storage, and use of biological data. Maintaining confidentiality is crucial to preserving trust in health databases and biobanks, particularly in the context of sensitive Women’s health data as planned in our study. The UAE has an ethical and legal framework for biobanks (36) that are consistent with international standards, including the European Union's General Data Protection Regulation (GDPR) and other relevant regulations and we intend to conduct our study adhering to these regulations. Like our local regulations in the UAE, countries, like Austria (34), have implemented specific legislative changes in response to evolving ethical considerations, such as pseudonymization and the use of data for scientific, historical, and statistical purposes.

This feasibility study is a starting point, not full-scale implementation. The pilot sample size may not capture the full diversity of conditions. Another potential challenge with establishing the biobank to support the Dubai Women’s Health Study lies in the infrastructure and logistical considerations of biobanks. Effective data management is essential to ensure the integrity, quality, and accessibility of biobank resources (37). This includes the use of ultra-low temperature (ULT) storage methods, such as ULT freezers and liquid nitrogen, which, while necessary for preserving biological samples, contribute to significant carbon emissions. However, in our study, samples will be stored at −80 °C and not in liquid nitrogen since it is not currently available at the Dubai Health Biobank. The Biobank comprises an Azenta BioStore automated storage system for managing and storing samples at −80 °C. The Azenta BioStore −80 °C automated storage systems use up to 75% less energy than equivalent traditional, ultra-low temperature (ULT) freezers.

The strengths of this feasibility study include recruitment from a large tertiary Women’s hospital, integration with an established biobank infrastructure, and alignment with national policy. Limitations include the single-site recruitment, limited follow-up duration, and the pilot sample size, which may not capture full population diversity. These findings will inform scaling to a larger cohort. Another limitation is the use of one-time dietary data collection methods, such as 24-h recalls. These can introduce measurement errors and bias in diet–disease assessments (18). This is a challenge shared by biobanks worldwide and highlights the need for repeated and more robust data collection methods to improve the accuracy of health outcomes derived from biobank data. This is a matter we intend to address in future studies.

Finally, one of the technical challenges identified in the biobank landscape is the lack of standardization (38) across different research domains. While reference standards exist in biobanks and digital repositories, ensuring the integration and reproducibility of data, especially in the -omic fields, remains an open challenge. To address this, it is important that efforts are directed towards standardization and the development of models, such as those using the JavaScript Object Notation (JSON) format, to integrate diverse data types from different domains. We have taken a step towards addressing this challenge in our study by initiating contact with the Endometriosis Phenome and Biobanking Harmonization Project (EPHect).

In conclusion, a dedicated Women’s biobank in Dubai will be critical for future translational research. It will address high-priority Women’s health issues such as endometriosis, PCOS, pre-eclampsia, and EC, which also contribute to the UAE's “Big 4” public health challenges (7). By investing in such an innovation, we could unlock new insights on the risk of disease processes, enhance efficacy of treatment protocols and eventually improve the quality of life of millions of women worldwide. Having a centralized repository of biological samples will aid in accelerating research and promote collaboration globally, among researchers, clinicians, and institutions, fostering innovation. Finally, we would be addressing a historical relative neglect in medical research on women which would reduce health inequities between genders, be beneficial globally and speed up progress towards achieving the United Nations Sustainable Development Goals.

## Ethics statement

The studies involving humans were approved by Institutional review board (IRB) approval from the MBRU (Mohamed Bin Rashid University) IRB in Dubai, obtained in February 2025 (MBRU IRB-2024-692). The studies were conducted in accordance with the local legislation and institutional requirements. The participants provided their written informed consent to participate in this study.
